# Supplementary material for: Cannabidiol Inhibits Tumorigenesis in Cisplatin-Resistant Non-Small Cell Lung Cancer via TRPV2
Source: Cancers (Basel). 2022 Feb 24;14(5):1181. doi: 10.3390/cancers14051181 (PMC8909073; doi:10.3390/cancers14051181)
Supplement: Supplementary file 1 [file cancers-14-01181-s001.zip › cancers-1577690-supplementary.pptx]

## Slide 1
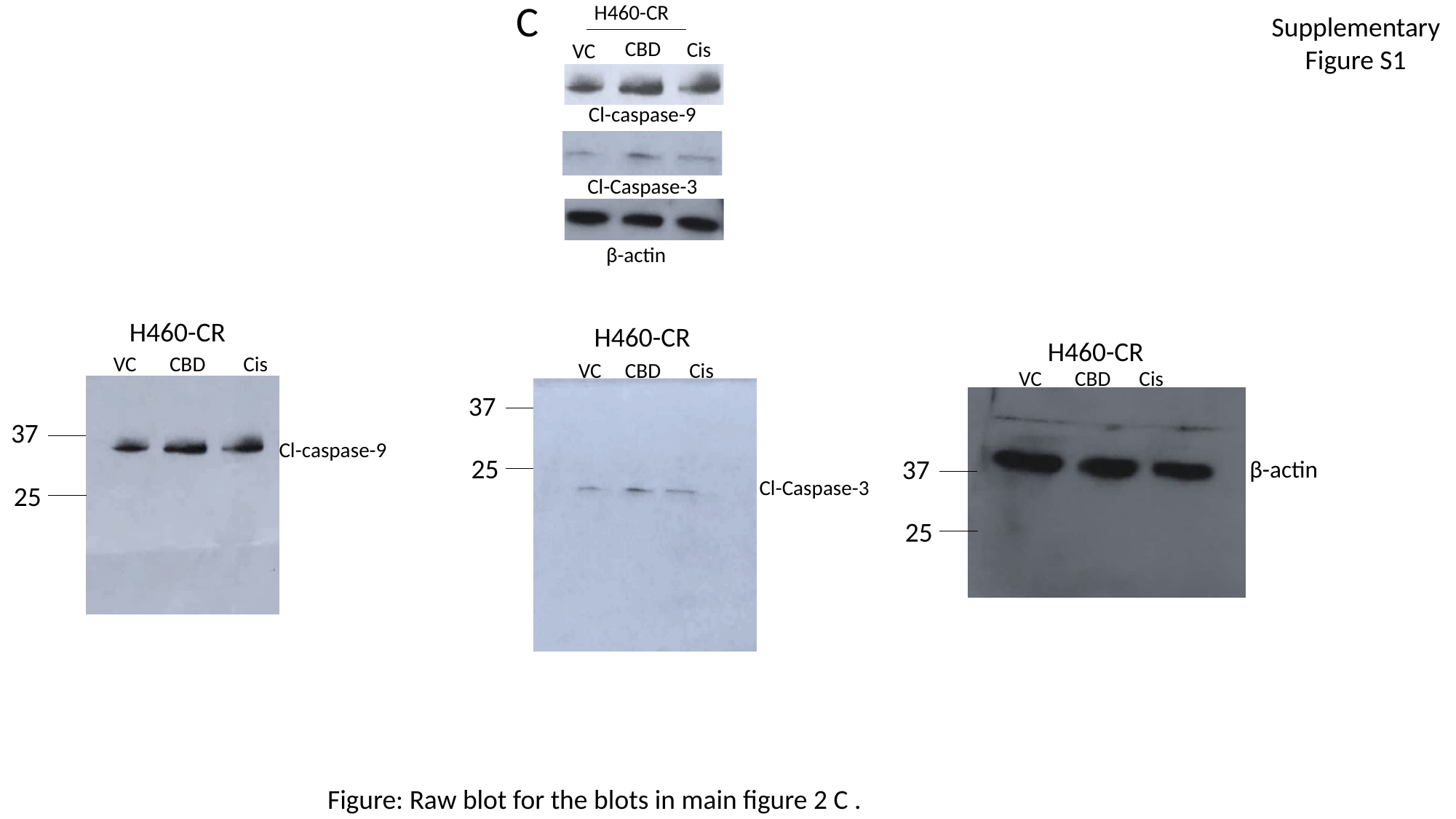

C
H460-CR
Supplementary Figure S1
CBD
Cis
VC
Cl-caspase-9
 Cl-Caspase-3
β-actin
H460-CR
H460-CR
H460-CR
VC CBD Cis
VC CBD Cis
VC CBD Cis
37
37
Cl-caspase-9
25
37
β-actin
 Cl-Caspase-3
25
25
Figure: Raw blot for the blots in main figure 2 C .

## Slide 2
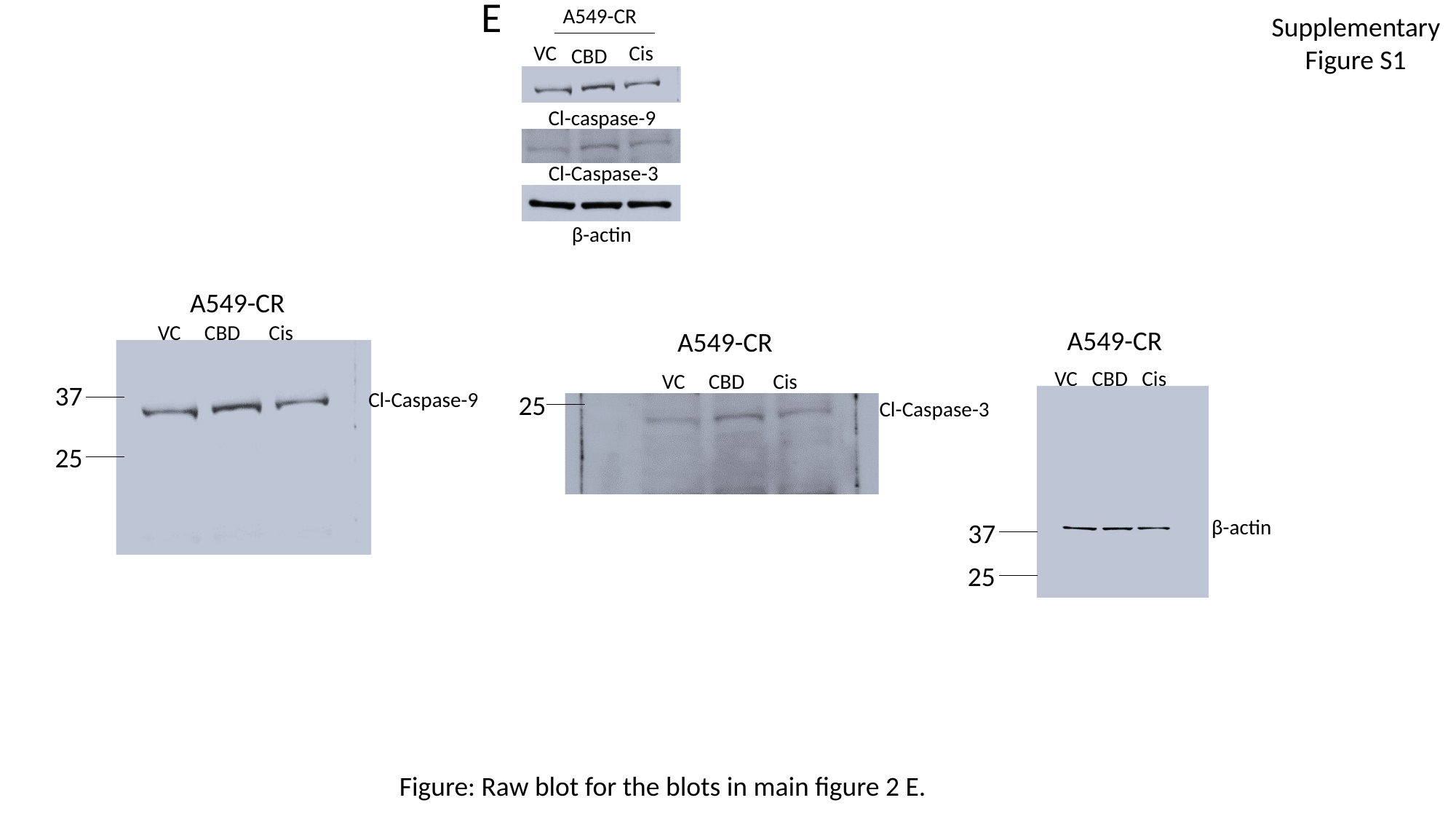

E
A549-CR
Cis
VC
CBD
Cl-caspase-9
 Cl-Caspase-3
β-actin
Supplementary Figure S1
A549-CR
VC CBD Cis
A549-CR
A549-CR
VC CBD Cis
VC CBD Cis
37
 Cl-Caspase-9
25
 Cl-Caspase-3
25
β-actin
37
25
Figure: Raw blot for the blots in main figure 2 E.

## Slide 3
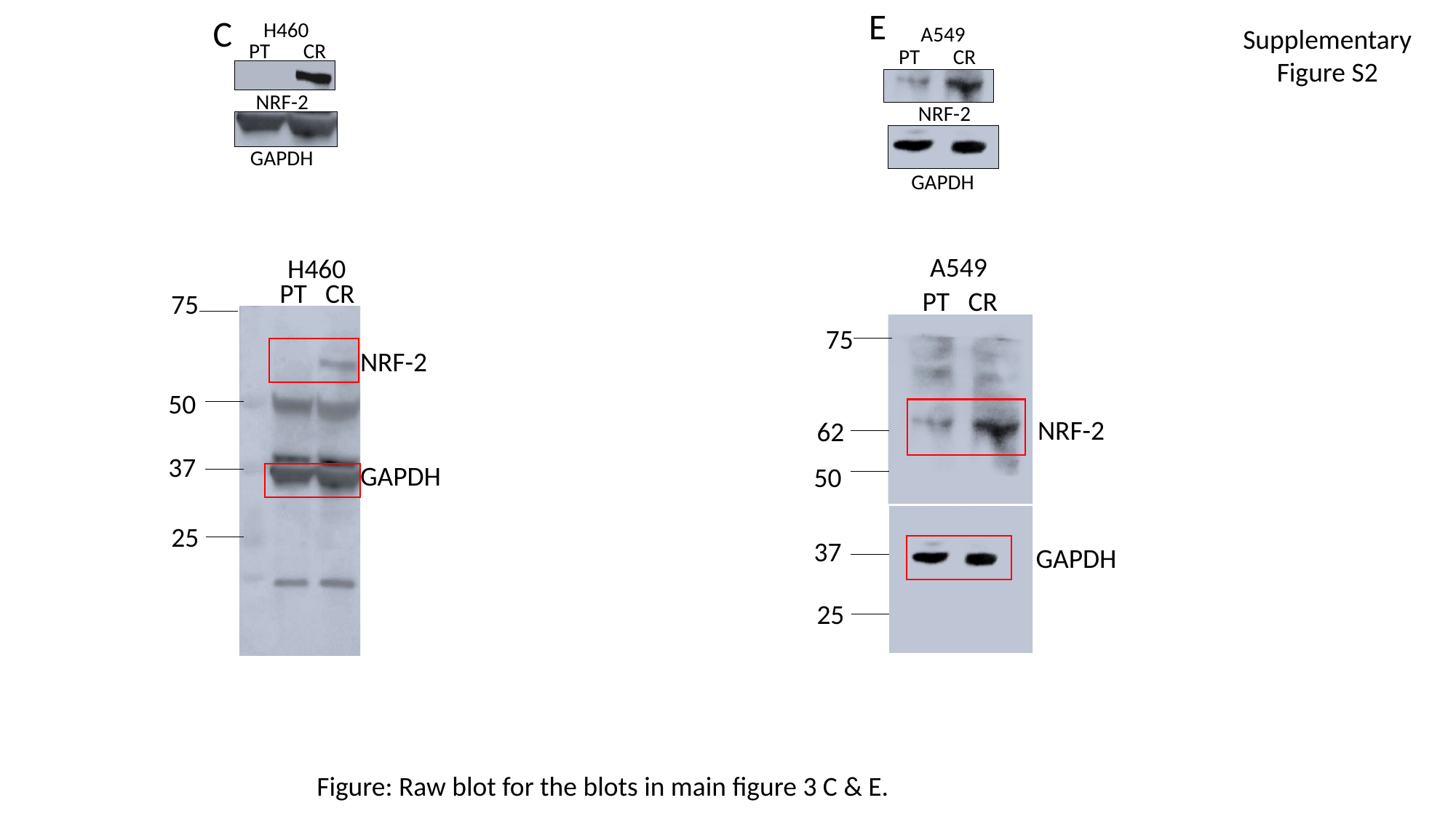

E
C
H460
A549
Supplementary Figure S2
PT CR
PT CR
NRF-2
NRF-2
GAPDH
GAPDH
A549
H460
PT CR
PT CR
75
50
37
GAPDH
25
75
NRF-2
NRF-2
62
50
37
GAPDH
25
Figure: Raw blot for the blots in main figure 3 C & E.

## Slide 4
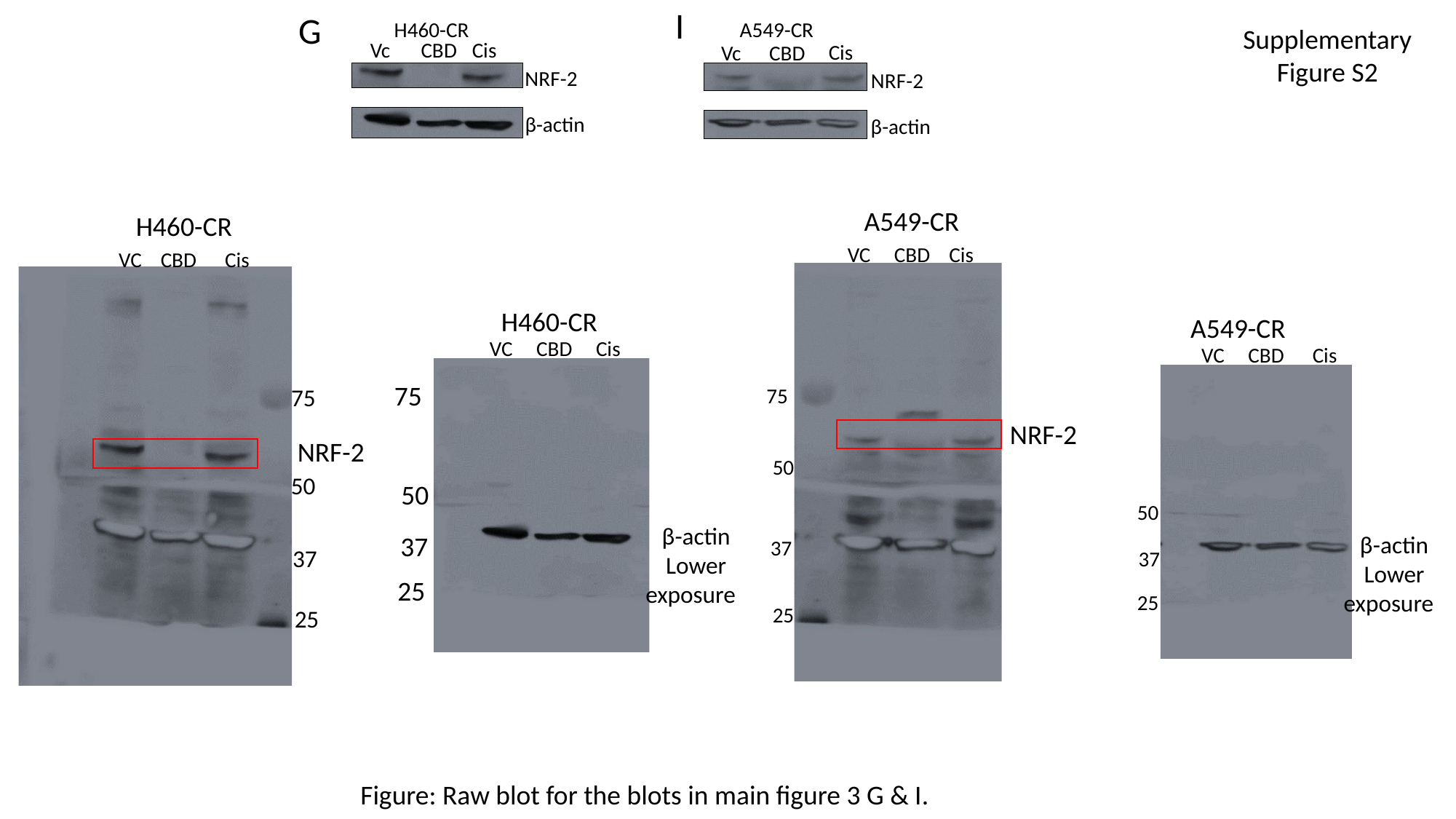

I
G
A549-CR
Cis
Vc
CBD
NRF-2
β-actin
H460-CR
Vc
CBD
Cis
NRF-2
β-actin
Supplementary Figure S2
A549-CR
H460-CR
VC CBD Cis
VC CBD Cis
H460-CR
A549-CR
VC CBD Cis
VC CBD Cis
75
75
75
NRF-2
NRF-2
50
50
50
50
β-actin
Lower exposure
37
β-actin
Lower exposure
37
37
37
25
25
25
25
Figure: Raw blot for the blots in main figure 3 G & I.

## Slide 5
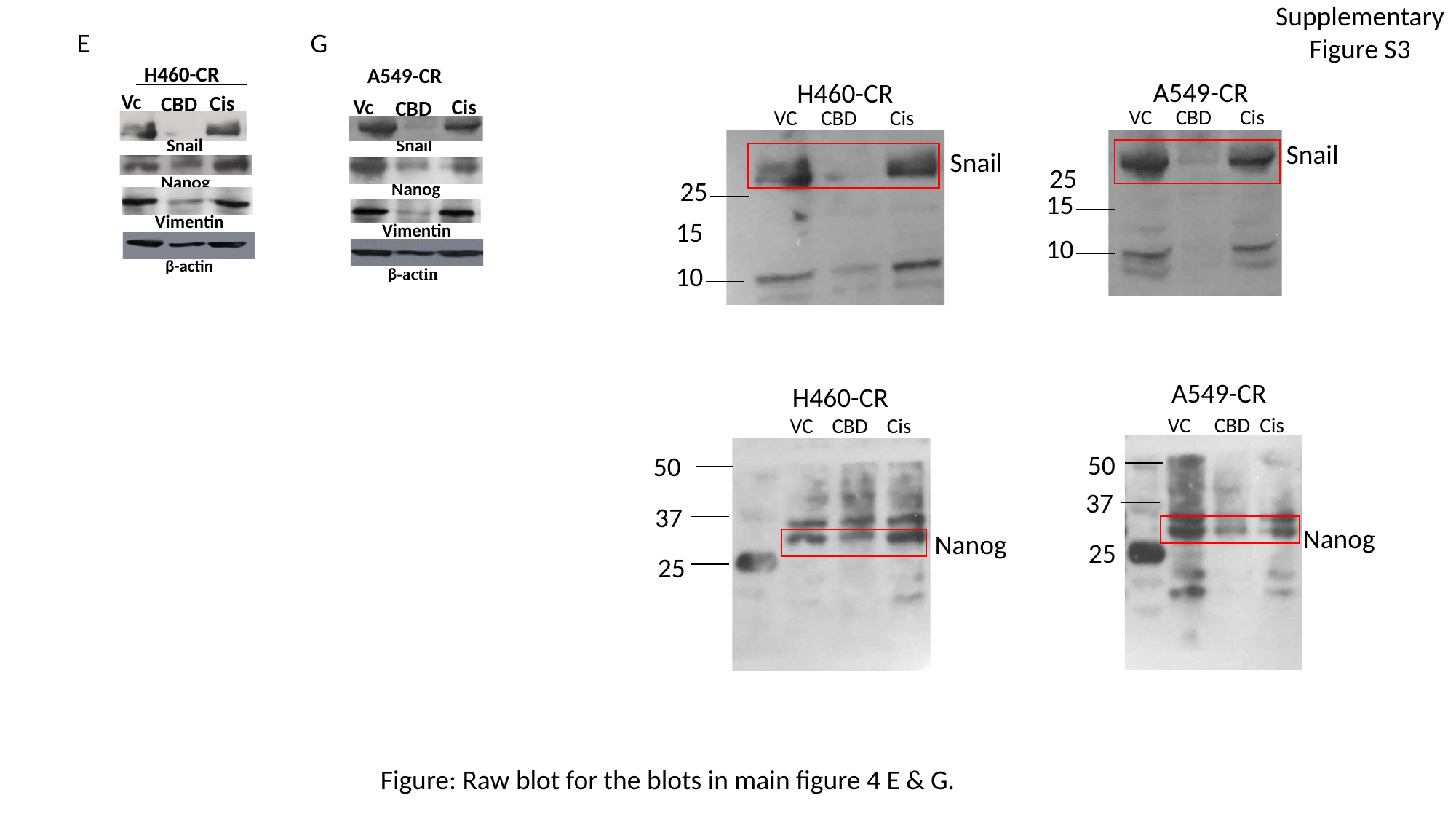

Supplementary Figure S3
E
G
H460-CR
A549-CR
A549-CR
H460-CR
Vc
Cis
CBD
Vc
Cis
CBD
VC CBD Cis
VC CBD Cis
Snail
Snail
Snail
Snail
25
Nanog
25
Nanog
15
Vimentin
15
Vimentin
10
β-actin
10
β-actin
A549-CR
H460-CR
VC CBD Cis
VC CBD Cis
50
50
37
37
Nanog
Nanog
25
25
Figure: Raw blot for the blots in main figure 4 E & G.

## Slide 6
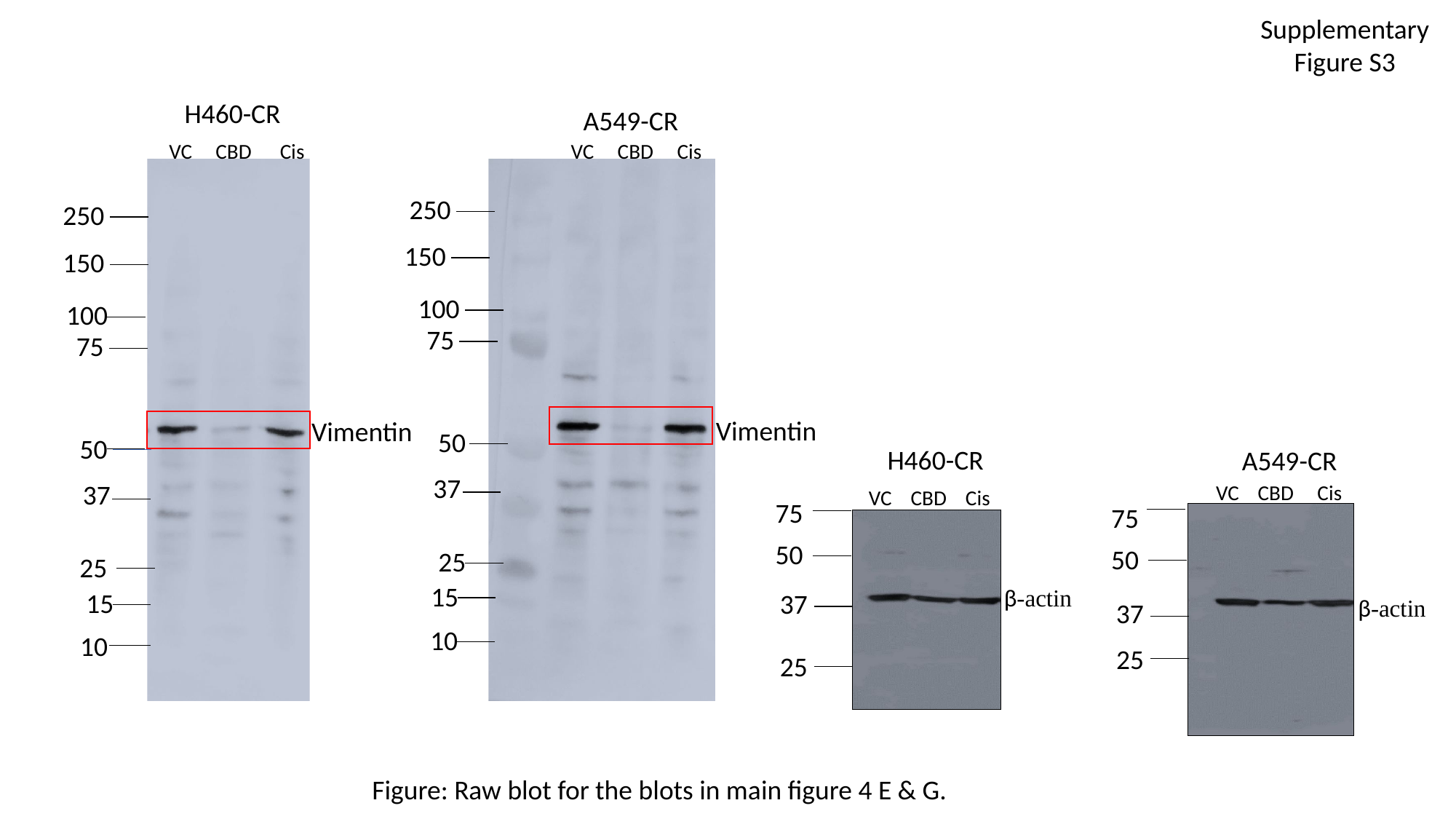

Supplementary Figure S3
H460-CR
A549-CR
 VC CBD Cis
VC CBD Cis
250
250
150
150
100
100
75
75
Vimentin
Vimentin
50
50
H460-CR
A549-CR
37
37
 VC CBD Cis
VC CBD Cis
75
75
50
50
25
25
15
β-actin
15
37
β-actin
37
10
10
25
25
Figure: Raw blot for the blots in main figure 4 E & G.

## Slide 7
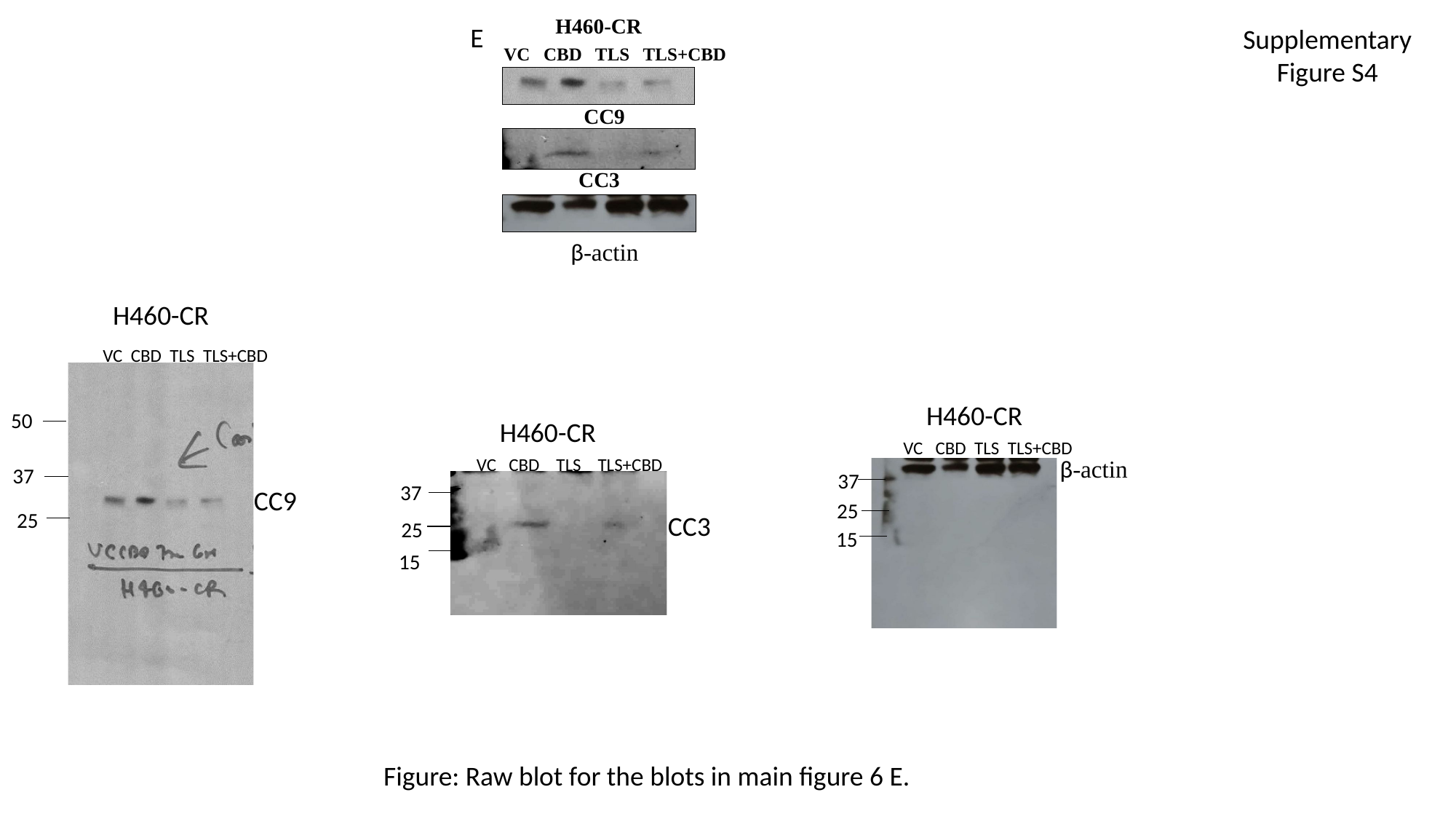

H460-CR
 VC CBD TLS TLS+CBD
CC9
CC3
E
β-actin
Supplementary Figure S4
H460-CR
 VC CBD TLS TLS+CBD
H460-CR
50
H460-CR
VC CBD TLS TLS+CBD
VC CBD TLS TLS+CBD
β-actin
37
37
37
CC9
25
25
CC3
25
15
15
Figure: Raw blot for the blots in main figure 6 E.

## Slide 8
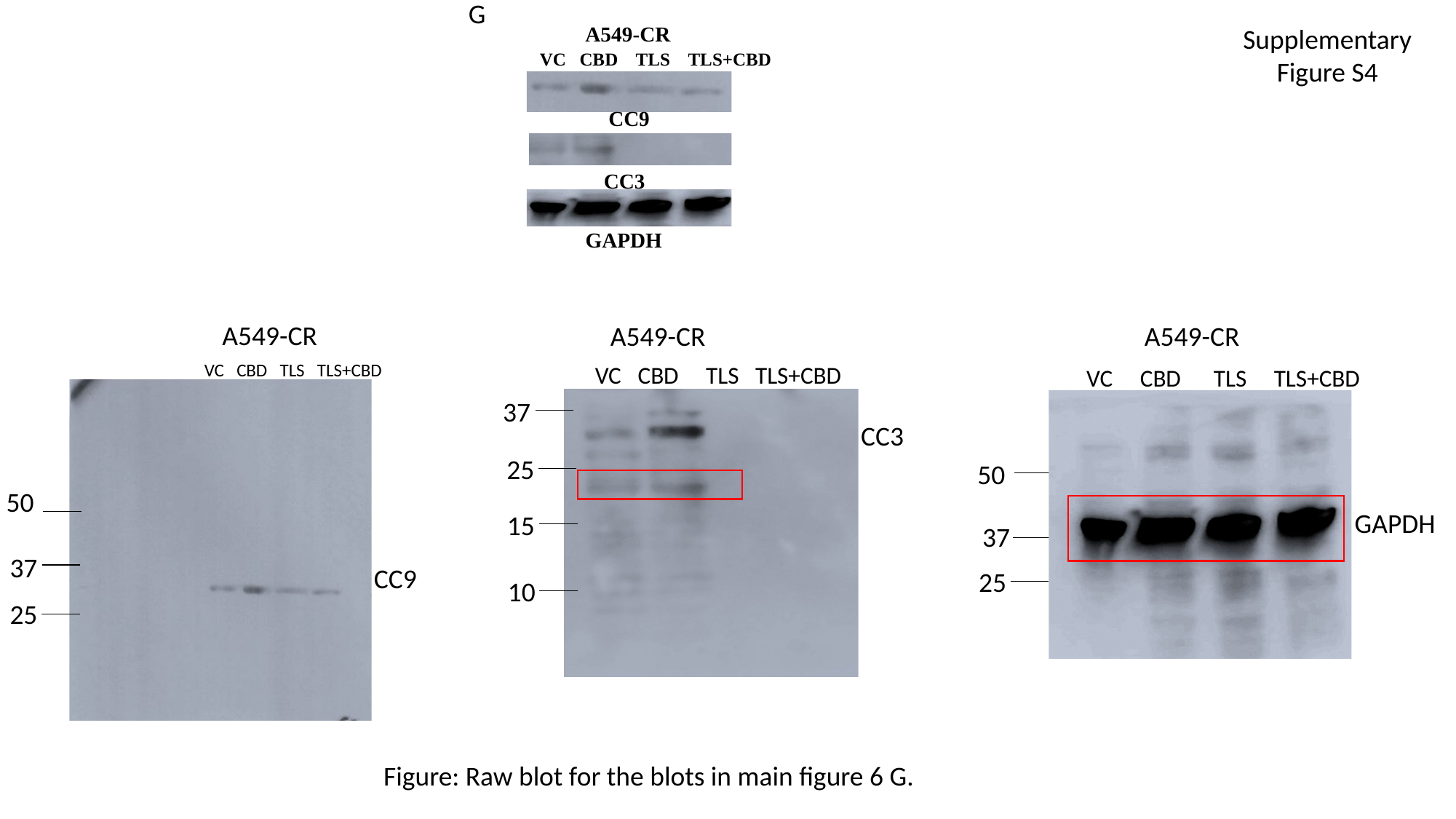

G
A549-CR
 VC CBD TLS TLS+CBD
CC9
CC3
GAPDH
Supplementary Figure S4
A549-CR
A549-CR
A549-CR
VC CBD TLS TLS+CBD
VC CBD TLS TLS+CBD
VC CBD TLS TLS+CBD
37
CC3
25
50
50
GAPDH
15
37
37
CC9
25
10
25
Figure: Raw blot for the blots in main figure 6 G.
